# Antifungal and antibacterial activities of polyherbal toothpaste against oral pathogens, in *vitro*

**DOI:** 10.18502/cmm.4.2.65

**Published:** 2018-06

**Authors:** Batool Sadeghi-Nejad, Eskandar Moghimipour, Sedigheh Yusef Naanaie, Shahrzad Nezarat

**Affiliations:** 1 Abadan School of Medical Sciences, Abadan, Iran; 2 Medicinal Plant Research Center, Faculty of Pharmacy, Ahvaz Jundishapur University of Medical Sciences, Ahvaz, Iran; 3 Agricultural and Natural Resources Center, Ahvaz, Iran; 4 Student Research Committee, Abadan School of Medical Sciences, Abadan, Iran

**Keywords:** Antibacterial, Antifungal, Oral pathogens, Polyherbal toothpaste, Yeast

## Abstract

**Background and Purpose:**

Herbal toothpastes are more secure and efficacious and less poisonous due to containing natural chemicals as compared with the synthetic toothpastes. The present study aimed to formulate a polyherbal toothpaste using accessible medicinal plants in Iran and evaluate its efficiency in the protection of oral hygiene and prevention of dental caries.

**Materials and Methods:**

The developed toothpaste was made of the leaf extracts of *Artemisia dracunculus*, *Satureja khuzestanica *(Jamzad), and* Myrtus communis *(Linn), combined at four different dilutions, namely 1:4 (25%), 1:1 (50%), 3:4 (75%), and (100%), with sterile distilled water. The product was tested against five microorganisms, including *Streptococcus mutans, Lactobaccilus caseie, S. sanguis, S. salivarius, *and* Candida albicans,* using agar well diffusion method.

**Results:**

After 24 h of incubation, the maximum mean diameters of inhibition zone against *L. caseie* and *C. albicans* were obtained as 17-30 and 10-25 mm, respectively. Furthermore, the minimum mean diameter of inhibition zone against *S. salivarious* was estimated as 15-20 mm.

**Conclusion:**

The formulated toothpaste showed potent inhibitory activities against Gram-positive bacteria and *C. albicans*. Therefore, more studies are required to accurately investigate the efficacy of the formulated toothpaste.

## Introduction

One of the most common chronic oral infections in the world is dental caries [1]. Oral pathogenic microorganisms have been the cause of dental plaques, dental caries, as well as gingival and periodontal diseases [2].* Streptococcus mutans *is one of the main opportunistic pathogens of dental caries, which is responsible for the formation of dental plaque and caries [3]. Other microorganisms associated with this oral condition include *Escherichia coli, S. aureus* [4], and *Candida *species. 

*C. albicans* is the most frequent yeast isolated from the oral cavities with poor oral hygiene [2]. The formulation ingredients of commercial toothpastes are mostly chemical substances, such as fluoride and whitening agents [2]. The literature contains evidence regarding the adverse effects of fluoride and bleaching agents (e.g., peroxide-based agents) used in the commercial toothpastes [5]. 

With this background in mind, the present study aimed to formulate a polyherbal toothpaste without any fluoride or whitening agents and evaluate its antimicrobial properties. The main ingredients of this toothpaste included the leaf extracts of *Artemisia dracunculus *L. (ADL), *Satureja khuzestanica *(Jamzad; SKJ), and* Myrtus communis *(Linn; MCL)*. *The MCL or myrtle, belonging to the Myrtaceae (Lamiaceae) family, is an aromatic evergreen small tree with small foliage and numerous branches [6]. In ancient medicinal herbs, myrtle leaves and flowers were used for the treatment of respiratory problems, dysentery, urinary tract infections, and candidiasis as a mouthwash [7]. 

According to the literature, the essential oil of MCL has pharmacological activities, including antioxidant [8], antimicrobial [9, 10], and antifungal activities [11, 12]. There is evidence regarding the inhibitory activity of the essential oil of MCL against clinically isolated oral pathogenic microorganisms [13]. *Artemisia *is a small and continual aromatic shrub from the Asteraceae family [14], which is called "Tarkhon" in Iran. In traditional medicine, this plant is used for the remedy of stomach pains, fever, and diabetes and is known to have anti-inflammatory, anti-parasitic, antioxidant, and antimicrobial activities [15]. 

On the other hand, SKJ*, *belonging to the Lamiaceae family, is extensively grown in the northern Khuzestan and southern Lorestan provinces of Iran [16]. This plant has traditionally been used for relieving tooth pain, strengthening the gum, and healing the wound in the southern part of Iran [17]. Moreover, this herb has been applied for antimicrobial [18] and antifungal [19] purposes, as well as the treatment of infectious diseases [20-21]. With regard to the previous studies reporting the antimicrobial properties of polyherbal toothpaste [22, 23], the current study was conducted to formulate a new polyherbal toothpaste containing the aqueous herbal extracts available in Iran and evaluate its antimicrobial potency against oral pathogens. 

## Materials and Methods

***Preparation of extracts***

The ADL was purchased from the local market, and MCL and SKJ were prepared with the aids of the Agricultural and Natural Resources Center, Ahvaz, Iran. The collected plants were air dried in shade. About 10 g of each powdered air dried plant was added to100 ml sterile distilled water (1:1 W/V) in a glass beaker for maceration, and then incubated on a rotary shaker for 72 h [24]. In the next step, the filtration of suspension was accomplished using Whatman filter paper No.1. The filtrated aqueous extracts were evaporated and dried at the room temperature. The extracts were stored in air-tight containers at -20˚C until future use.

***Microorganisms and inoculum preparation***

The antimicrobial activity of four anaerobic bacteria (Gram-positive) isolated from clinical isolates and *Candida *species was assessed. A total of twelve anaerobic bacteria, including* S. sanguis, S. salivarius, S. mutans, *and* L. casei,* were prepared from the frozen stock cultures obtained from the Department of Medical Microbiology, Ahvaz Jundishapur University of Medical Sciences, Ahvaz, Iran. In addition, five *C. albicans* strains were isolated from patients with periodontitis and gingivitis referring to the Educational Clinics of Dentistry School, Ahvaz Jundishapur University of Medical Sciences, Ahvaz, Iran. 

These samples were subcultured, and then diluted in a sterile normal saline solution (0.9%) to obtain a concentration of 5×10^5 ^CFU/ml for fungal strains and a colony forming unit of 10^6^ (CFU/ml) for bacterial strains, adjusted with the turbidity of 0.5 McFarland [25, 26]. 

***Formulation of polyherbal toothpaste***

The ingredients of the fluoride-free polyherbal toothpaste was prepared according to the procedure adopted by Sekar and Zulhilmi Abdullah [22] with some modifications (Table 1).

***Screening polyherbal toothpaste for antimicrobial activity***

The formulation of the toothpaste was accomplished using three plants, namely ADL, MDL, and SKL, which were previously confirmed to have antimicrobial activities by in vitro assays. The dentifrice solution was prepared according to the previously reported procedures [2, 27]. 

The solution was tested against five microorganisms, including* S. mutans, L. caseie, S. sanguis, S. salivarius, *and* C. albicans,* by using agar well diffusion method following the previous studies [28, 29]. The agar plates inoculated with bacteria were kept in an anaerobic cabinet supplied with CO_2_ at 37°C for 24, 48, and 72 h. On the other hand, those agar plates inoculated with *C. albicans* were incubated at 30°C for 48 h [30].

***Statistical analysis***

Statistical analysis was performed in SPSS software (version 20.0). The mean diameters of the inhibition zones were calculated. P-value less than 0.05 was considered statistically significant.

***Quality parameters of formulated polyherbal toothpaste***

The organoleptic investigation of polyherbal toothpaste, including color, taste, odor, and texture, were carried out by sensational and visual surveys according to the modified procedure of Sekar and Zulhilmi Abdullah [22].

**Table 1:** Ingredients of the formulated polyherbal toothpaste

**Components **	**Amounts g/%**	**Property**
*A. dracunculus *leaf extract	0.0625	Active ingredient
*S. khuzestanica *leaf extract	0.0625	Active ingredient
*M. communis *leaf extract	0.0625	Active ingredient
Hydroxypropyl methyl cellulose	3	Gelling agent
Sodium lauryl sulphate	5	Surfactant
Calcium carbonate	25	Abrasive
Glycerin	5	Anti-crusting agent
Methyl paraben	0.5	Preservative
Propyl paraben	0.25	Preservative
Sodium saccharine	0.3125	Sweetener
Peppermint oil	0.75 (2-3 drops)	Flavoring agent
Demineralized water	60.5	Vehicle
Total	100 ml	

## Results

**Figure 1:**
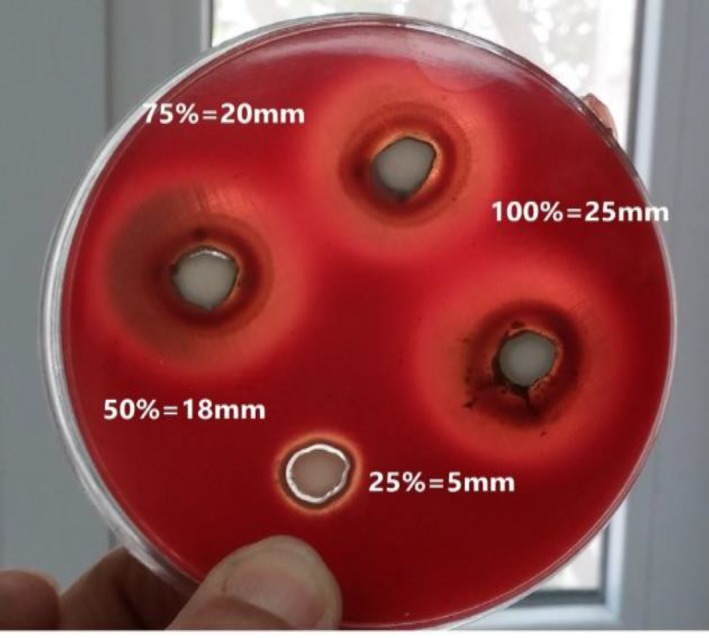
Inhibition zones increased by polyherbal toothpaste against

**Figure 2:**
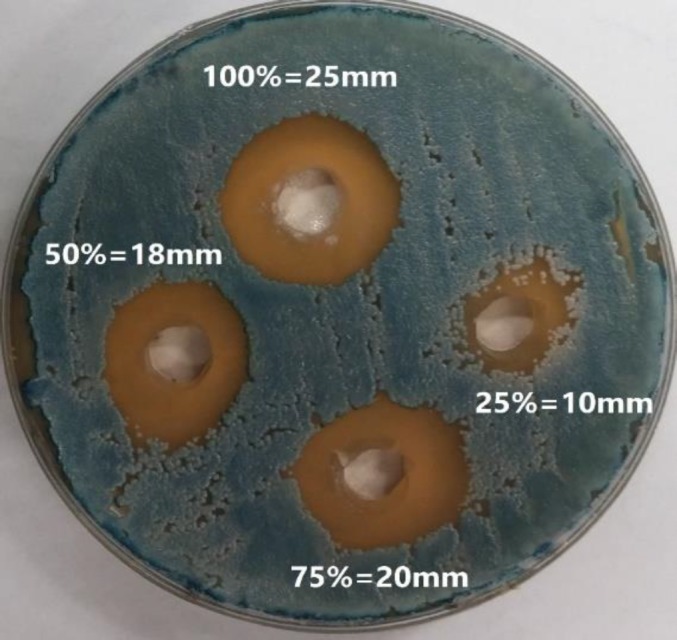
Inhibition zones increased by polyherbal toothpaste against *Candida albicans *on Sabouraud dextrose agar at four different dilutions

Table 2 summarized the inhibition zones produced by four polyherbal toothpaste dilutions of full strength, 3:4, 1:1, and 1:4 against *S. mutans, S. salivarius, S. sanguis, L. casei,* and* C. albicans*. The mean values of the microbial inhibition zones are shown in Table 2. The results demonstrated that *L. casei* showed the highest sensitivity against the dilutions of polyherbal toothpaste ranging from 5-30 mm in 24 h, followed by *C. albicans *(10-25 mm), *S. mutans* (5-25 mm),* S. salivarius* (10-25 mm), and *S. sanguis *(5-20 mm) at different dilutions of the toothpaste (figures 1 and 2; Table 2). All dilutions of polyherbal toothpaste were effective in inhibiting the growth of the tested bacterium and fungus; however, they had no inhibitory effect on *L. caseie*, *S. sanguis,* and *S. mutans* at the dilution of 1:4. 

The polyherbal toothpaste showed a significant antimicrobial activity against all tested bacterium and yeast (*P<0.001*). Table 3 tabulates the results of one-sample t-test for each dilution of toothpaste against all the tested strains. The comparison of antimicrobial activities against tested microorganism in different concentrations is demonstrated in Table 4.

Table 5 exhibits the results of the physicochemical parameters of the formulated toothpaste.

**Table 2:** Antimicrobial activity of polyherbal toothpaste against five dental caries pathogens by agar well diffusion method

**Mean diameter of growth inhibition zones (mm)**					
**Dilutions of** **pt**	***S. salivarius***	***S. sanguis***	***S. mutans***	***L. casei***	***C. albicans***
1:4 (25%)	^a^12.33**±**2.51	3.33**±**1.52	3.33**±**2.08	4.00**±**1.00	7.33**±**2.51
1:1 (50%)	18.33**±**1.52	12.33**±**2.51	15.00**±**3.00	17.33**±**2.51	15.00**±**3.00
3:4 (75%)	19.00**±**3.60	15.00**±**3.00	17.66**±**2.51	24.00**±**3.60	17.33**±**2.51
FS (100%)	20.66**±**4.04	17.66**±**20.51	21.00**±**3.60	25.00**±**5.00	20.00**±**5.00

PT: polyherbal toothpaste, FS: full strength

a Values including the diameter of the well (7 mm) are means of three replicates.

*Streptococcus mutans *on blood agar at four different dilutions

**Table 3:** Results of one-sample t-test for each dilution of toothpaste against all tested strains

**Dilution of polyherbal toothpaste**	**Mean±SD**	**Significant (2-tailed)**
1:4 (25%)	6.06±3.97	*P<0.05 0.001*
1:1 (50%)	15.60±3.06	*P<0.05 0.001*
3:4 (75%)	18.60±4.04	*P<0.05 0.001*
FS (100%)	20.86±4.27	*P<0.05 0.001*

Significance level: < 0.05, FS: full strength

**Table 4:** Comparison of antimicrobial activity against tested microorganisms in different concentrations

**Dilution of polyherbal toothpaste**	**Mean±SD **	**Significant (2-tailed)**
Pair 1 Microbs-PMT 1:4 (25%)	3.66±4.48	*P<0.05 0.007*
Pair 2 Microbs-PMT 1:1 (50%)	13.20±3.46	*P<0.050 0.001*
Pair 3 Microbs-PMT 3:4 (75%)	16.20±4.12	*P<0.050 0.001 *
Pair 4 Microbs-PMT FS (100%)	18.46 ±4.32	*P<0.050 0.001*

Significance level: < 0.05, FS: full strength

**Table 5:** Organoleptic investigation of the formulated polyherbal toothpaste

**Parameter **	**Result**
Color	Bright green
Taste	Slightly Bitter
Odor	Mint
Texture	Smooth
Moisture content	39%
Foaming identity	23 ml
pH	8
Storage constancy	After 45 days, no observation was made regarding the separation of the liquid and solid phases of the toothpaste.

## Discussion

A good oral hygiene maintenance is the key to prevent oral diseases. The novelty of the herbal toothpaste developed in the current study owes to its natural compounds as aqueous extract. Therefore, it can be claimed that this toothpaste can be used as a completely natural product without the complications of the commercial products. The results revealed that our developed toothpaste had different degrees of effectiveness against the five tested microorganisms. In this regard, the formulated toothpaste exerted a highly significant effect against all tested oral pathogens. 

As the results indicated, *L. caseie *and *S. sanguis* had the highest and lowest sensitivities to the formulated polyherbal toothpastes, exhibiting inhibition zones of 17-30 and 15-20 mm, respectively. In a clinical study, a commercially available toothpaste was reported to have inhibitory effects on some pathogenic oral microorganisms, such as *S. mutans*, *Micrococcus *species, *Proteus vulgaris, S. aureus, *and *C. albicans* [29]. 

There are a number of polyherbal toothpastes playing an important role in dental prophylaxis and improvement of oral health [31]. In a study conducted by Sekar and Zulhilmi Abdullah, *S. aureus *was reported as the most sensitive species to polyherbal toothpaste with an inhibition zone of 10-15 mm, followed by *E. coli *(9-12 mm), *Bacillus cereus *(7-12 mm), and *Pseudomonas aeruginosa *(9-11 mm) [22]. In another study, the polyherbal dentifrices containing Neem, Pudina, Long, Babul, Turmeric, and Vajradanti showed significant antimicrobial activities against *E. coli*, *S. aureus*, *S. mutans, *and C*. albicans *[23]. 

In a study conducted by Sharma et al. (2014), the methanolic extract of polyherbal formulation showed the highest and lowest activities against *S. mutans *and *C. albicans, *respectively [32]. In the present study, the formulated toothpaste demonstrated a good activity against* C. albicans* in various concentrations (i.e., 100%, 75%, 50%, and 25%) and exhibited a strong activity against *L. caseie, S. mutans,* *S. sanguis, *and *S. salivarius* in 100%, 75%, 50% concentrations. 

Moreover, the presence of carvacrol (2-methyl-5-[1-methylethyl]-phenol) (96.9%) as the main component of *S. khuzestanica* essential oils (SKEO) may be responsible for the antimicrobial properties of the present toothpaste. It was also reported that the SKEO has remarkable antibacterial effects, particularly against the resistant *S. aureus* [33]. In another study, *S. khuzestanica *leaf extract was reported to show a significant antibacterial activity against* S.* *mutans* in the range of 300-500 µg/ml [34]. 

Zomorodian et al. investigated the antimicrobial activity of seven essential oils against the common species accounting for oral infections, such as *S. mutants, S. sanguis, S. salivarius, E. faecalis, S. aureus, C. albicans C. glabrata*, *C. dubliniensis, *and* C. krusei*. The results of the mentioned study revealed that SKJ and *A. sieberi* were active against all tested oral pathogens. In addition, in the mentioned study, SKJ showed the highest antimicrobial activities, while *A. sieberi *exhibited the lowest antimicrobial activity [35]. 

The consistency between the results of the aforementioned study and those of our research can be due to the utilization two plants used in the mentioned research for the formulation of our polyherbal toothpaste. Moreover, there is evidence confirming the antimicrobial effects of the essential oil of MCL on clinically isolated oral pathogens. All isolates were sensitive to MCL at 125-1000 μg/ml by agar disk diffusion with inhibition zones of 8.1-41.25 mm in diameter. Furthermore, all *S. pyogenes*, *S. mutans, *and *C. albicans *strains showed sensitivity at 62.5 μg/ml. 

The* S. mutans*, as the main etiological agent of dental caries, has been recorded as the second most sensitive pathogen to MCL (21). Previous studies have also reported that the main compounds of the essential oil of MCL are terpenoid compounds (i.e., 1,8- cineole, α-pinene, myrtenyl acetate, limonene, linalool, and α-terpinolene), flavonoids and tannin that are responsible for the inhibitory effects of the MCL in the formulated toothpaste [36]. 

In addition, Sharafati Chaleshtori demonstrated that the essential oil of ADL has an antibacterial activity against *S. aureus, Alcaligenes faecalis*, *Providencia rettgeri*, *Serratia marcescens*, *Shigella dysenteriae*, *Listeria monocytogenes,* and *Klebsiella oxytoca *[37]. Based on these observations, the efficiency of polyherbal toothpaste can be ascribed to the presence of variant phytochemical compounds in plant extracts. 

The novelty of the present study is the use of the aqueous extracts of selected plants instead of chemical solvents, such as acetone, ethanol, and methanol. Moreover, the extracts utilized in our polyherbal toothpaste had no side effects as confirmed by an *in vivo* study previously performed by the authors of the current study.

## Conclusion

Further studies are recommended to make it as one of the commercial herbal toothpaste for the treatment of oral microbial infections.

## References

[1] Patro BK, Ravi Kumar B, Goswami A, Mathur VP, Nongkynrih B (2012). Prevalence of dental caries among adults and elderly in an urban resettlement colony of New Delhi. Indian J Dent Res.

[2] Prasanth M (2011). Antimicrobial efficacy of different toothpastes and mouthrinses: an in vitro study. Dent Res J.

[3] Gamboa F, Estupinan M, Galindo A (2004). Presence of Streptococcus mutans in saliva and its relationship with dental caries: antimicrobial susceptibility of the isolates. Univ Sci.

[4] Bagg J, Sweeney MP, Wood K, Wiggins A (1995). Possible role of Staphylococcus aureus in severe oral mucositis among elderly dehydrated patients. Microb Ecol Health Dis.

[5] Peters P, Drummond C (2013). Perioral dermatitis from high fluoride dentifrice: a case report and review of literature. Aust Dent J.

[6] Berka-Zougali R, Ferhat MA, Hassani A, Chemat F, Allaf KS (2012). Comparative study of essential oil extracted from Algerian Myrtus communis oil L leaves using microwaves and hydrodistillation. Int J Mol Sci.

[7] Mimica-Dukic N, Bugarin D, Grbovic S, Mitić-Culafić D, Vuković-Gacić B, Orcić D (2010). Essential oil of Myrtus communis L as a potential antioxidant and antimutagenic agents. Molecules.

[8] Belmimoun A, Meddah B, Meddah AT, Sonnet P (2016). Antibacterial and antioxidant activities of the essential oils and phenolic extracts of Myrtus communis and Zygophylum album from Algeria. J Fundam Appl Sci.

[9] Yadegarinia D, Gachkar L, Rezaei MB, Taghizadeh M, Astaneh SA, Rasooli I (2006). Biochemical activities of Iranian Mentha piperita and Myrtus communis L essential oils. Phytochemistry.

[10] Touaibia M (2015). Antimicrobial activity of the essential oil of Myrtus communis L berries growing wild in Algeria. J Fumdam Appl Sci.

[11] Sadeghi Nejad B, Erfani Nejad M, Yusef Naanaie S, Zarrin M (2014). Antifungal efficacy of Myrtus communis Linn. Jentashapir J Health Res.

[12] Mohammadi R, Mir HE, Shadzi SH, Moatar F (2008). Antifungal activity of Myrtus communis L essential oil against clinical isolates of Aspergillus. J Isfahan Med Sch.

[13] Fani MM, Kohanteb J, Araghizadeh A (2014). Inhibitory activity of Myrtus communis oil on some clinically isolated oral pathogens. Med Princ Pract.

[14] Weinoehrl S, Feistel B, Pischel I, Kopp B, Butterweck V (2012). Comparative evaluation of two different Artemisia dracunculus L cultivars for blood sugar lowering effects in rats. Phytother Res.

[15] Behbahani BA, Shahidi F, Yazdi FT, Mortazavi SA, Mohebbi M (2017). Antioxidant activity and antimicrobial effect of tarragon (Artemisia dracunculus) extract and chemical composition of its essential oil. J Food Measur Charact.

[16] Jamzad Z (2010). A new species of the genus Satureja (Labiatae) from Iran. Iran J Bot.

[17] Seghatoleslami S, Samadi N, Salehnia A, Azimi S (2009). Antibacterial activity of endemic Satureja Khuzistanica Jamzad essential oil against oral pathogens. Iran Endod J.

[18] Eftekhar F, Ashoori N, Yousefzadi M (2017). In-Vitro antimicrobial activity and chemical composition of S khuzestanica jamzad essential oils against multidrug-resistant Acinetobacter baumannii. Avicenna J Clin Microbiol Infect.

[19] Sadeghi-Nejad B, Shiravi F, Ghanbari S, Alinejadi M (2007). Antifungal activity of Satureja khuzestanica (Jamzad) leaves extracts. Jundishapur J Microbiol.

[20] Zargari A (2017). Medicinal plants.

[21] Momtaz S, Abdollahi M (2010). An update on pharmacology of Satureja species; from antioxidant, antimicrobial, antidiabetes and antihyperlipidemic to reproductive stimulation. Int J Pharmacol.

[22] Sekar M, Zulhilmi Abdullah M (2016). Formulation, evaluation and antimicrobial properties of polyherbal toothpaste. Int J Curr Pharm Res.

[23] Anushree B, Fawaz MA, Narahari R, Shahela T, Syed A (2015). Comparison of antimicrobial efficacy of Triclosan- containing, herbal and homeopathy toothpastes- an in vitro study. J Clin Diagn Res.

[24] Sadeghi-Nejad B, Azish M (2013). In vitro antibacterial and antifungal effect of some medicinal plants. Afr J Microbiol Res.

[25] Baron EJ, Finegold SM (2017). Diagnostic microbiology.

[26] Clinical and Laboratory Standards Institute (2017). Reference method for broth dilution antifungal susceptibility testing of filamentous fungi; approved standard (M38-A2).

[27] Rai Aneja K, Joshi R, Sharma C (2010). The antimicrobial potential of ten often used mouthwashes against four dental caries pathogens. Jundishapur J Microbiol.

[28] Shialy Z, Zarrin M, Sadeghi Nejad B, Yusef Naanaie S (2015). In vitro antifungal properties of Pistacia atlantica and olive extracts on different fungal species. Curr Med Mycol.

[29] Jabuk SI, AL-Harmoosh RA, Ghani Jabuk NA (2016). The antimicrobial effect of commercial available local brand of toothpastes against some dental caries microorganisms. Magazine Al-Kufa Univ Biol.

[30] Falahati M, Sepahvand A, Mahmoudvand H, Baharvand P, Jabbarnia S, Ghojoghi A (2015). Evaluation of the antifungal activities of various extracts from Pistacia atlantica Desf. Curr Med Mycol.

[31] Biswas G, Anup N, Acharya S, Kumawat H, Vishnani P, Tambi S (2014). Evaluation of the efficacy of 02% chlorhexidine versus herbal oral rinse on plaque induced gingivitis-a randomized clinical trial. J Nurs Health Sci.

[32] Sharma S, Agarwal1 SS, Prakash J, Pandey M, Singh A (2014). Formulation development and quality evaluation of polyherbal toothpaste “Oral S”. Int J Pharm Res Allied Sci.

[33] Hadavand S (2017). The carvacrol level and antimicrobial properties of industrial and laboratory essential oil of the wild and cultivated Satureja khuzestanica. BMJ Open.

[34] Taebi S, Nosrati M (2017). Evaluation of anti-bacterial activity and biofilm inhibition of Satureja Khuzestanica Jamzad against Streptococcus Mutans. Arak Univ Med J.

[35] Zomorodian K, Ghadiri P, Saharkhiz MJ, Moein MR, Mehriar P, Bahrani F (2015). Antimicrobial activity of seven essential oils from Iranian aromatic plants against common causes of oral infections. Jundishapur J Microbiol.

[36] Alipour G, Dashti S, Hosseinzadeh H (2014). Review of pharmacological effects of Myrtus communis L and its active constituents. Phytother Res.

[37] Chaleshtori RS, Rokni N, Razavilar V, Kopaei R (2013). The evaluation of the antibacterial and antioxidant activity of tarragon (Artemisia dracunculus L) essential oil and its chemical composition. Jundishapur J Microbiol.

